# Syndesmosis ankle injury in football players: a pilot study

**DOI:** 10.1186/1757-1146-5-S1-O53

**Published:** 2012-04-10

**Authors:** Amy Sman, Claire Hiller, Leslie Nicholson, Katherine Rae, Kathryn Refshauge

**Affiliations:** 1 Department of Physiotherapy, University of Sydney, Sydney, NSW, 2137, Australia; 2 The Sports Clinic, University of Sydney, Sydney, NSW, 2006, Australia

## Background

Syndesmosis ankle injury, also known as ‘high’ ankle sprain, appears to be increasingly common [1]. The injury occurs most commonly in high impact contact sports [2] resulting in greater impairment and longer rehabilitation than the typical ankle sprain [3,4]. However, no prospective study has investigated predictors and prognostic factors for syndesmosis injury.

## Materials and methods

We recruited football players from Sydney University Rugby Union and AFL clubs. The only exclusion criterion was symptoms at the time of screening that would affect baseline testing performance. Trained assessors conducted pre-season screening and participants were then followed for the season. The suite of baseline tests included vertical jump, star excursion balance test, single leg triple hop for balance and heel rise test. History of ankle injury and competition level was also recorded. Players who sustained an ankle injury during the season were retested within 2 weeks of injury or on removal of the boot, and when returned to play. Re-testing involved the weight bearing lunge, vertical jump, star excursion balance and a fear avoidance beliefs questionnaire.

## Results

During the 2011 season 102 players were screened, with 22 sustaining an ankle injury (13 lateral, 7 syndesmosis and 2 medial ankle sprains confirmed by MRI). Pilot results suggest that vertical jump performance together with a history of ankle sprain could be predictors for syndesmosis injury. Lower vertical jump height was highly correlated with longer recovery (r= -.571, p= .007). On average, recovery from syndesmosis injury took 85 days and lateral ankle sprains took 21 days. A difference of 13 cm was found between vertical jump following syndesmosis injury (32cm) and lateral ankle sprains (49cm).

## Conclusions

Sports medical staff and clinicians can use the results of this pilot study for screening and rehabilitation purposes. However, further study with a larger cohort is required and underway.
